# Abnormal Functional Network Centrality and Causal Connectivity in Migraine Without Aura: A Resting‐State fMRI Study

**DOI:** 10.1002/brb3.70414

**Published:** 2025-03-13

**Authors:** Di Zhang, Liyan Lu, Xiaobin Huang, Xiaojing Zhao, Yamei Zhang, Tong Fu, Fengfang Li, Xinying Wu

**Affiliations:** ^1^ Department of Radiology Nanjing First Hospital, Nanjing Medical University Nanjing Jiangsu Province China

**Keywords:** migraine without aura, degree centrality, Granger causality analysis, resting‐state fMRI

## Abstract

**Objective:**

The pathophysiological mechanism of migraine is still not clear. Thus, this study aimed to evaluate the changes in effective connectivity (EC) in the brain functional network underlying migraine and its association with clinical measures of migraine.

**Background:**

Fifty patients with episodic migraine without aura (MwoA) and 48 healthy controls (HCs) were enrolled in this study. Spontaneous activity in the brain was evaluated using the degree centrality (DC) method, and the brain regions with obvious signal differences between the two groups were taken as seed points for whole brain Granger causality analysis (GCA) analysis. The values of the brain regions with differences in DC and GCA were extracted and correlated with clinical measures of migraine.

**Results:**

Compared to the HCs, the MwoA patients showed decreased DC in the left inferior temporal gyrus (ITG) and increased DC in the right precuneus and exhibited significantly decreased EC from the left ITG to the left inferior parietal gyrus and right inferior occipital gyrus (IOG) as well as significantly increased EC from the left postcentral gyrus and left cerebellum posterior lobe to the left ITG. Moreover, decreased EC from the left thalamus to the right precuneus was found in the MwoA patients compared to the HCs. The DC values in the right precuneus were significantly negatively correlated with the duration of headache. Additionally, we found a significantly positive correlation between the Migraine Disability Assessment questionnaire score and the EC from the left ITG to the right IOG, as well as between the intensity of headache and the EC from the left thalamus to the right precuneus.

**Conclusions:**

This study found changes in the EC of the brain functional network underlying migraine and their associations with migraine‐related parameters. These findings are helpful for understanding the pathophysiological mechanism in migraine patients.

## Graphical abstract

The spatial distribution of degree centrality (DC) in migraine patients and normal controls was highly limited to the temporal and occipital lobes, anterior cuneus, prefrontal cortex, corpus callosum, and parahippocampal gyrus. Compared to the normal controls, the migraine patients showed decreased DC in the left inferior temporal gyrus (ITG) and increased DC in the right precuneus. Then, the ITG and right precuneus were used as seed points for Granger causality analysis. Decreased effective connectivity from the left ITG to the left inferior parietal gyrus and right inferior occipital gyrus, increased effective connectivity from the left postcentral gyrus and left cerebellum posterior lobe to the left ITG, and decreased effective connectivity from the left thalamus to the right precuneus can be found.

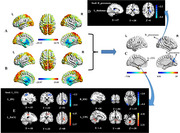

**Abbreviations**
CPG: cerebellum posterior lobe
DC: degree centrality
EC: effective connectivity
GCA: Granger causality analysis
GRF: Gaussian random field
HCs: healthy controls
HIT‐6: Headache Impact Test‐6
IOG: inferior occipital gyrus
IPG: inferior parietal gyrus
ITG: inferior temporal gyrus
MIDAS: Migraine Disability Assessment
MoCA: Montreal Cognitive Assessment
MwoA: migraine without aura
PoCG: postcentral gyrus
SAS: Self‐Rating Anxiety Scale
SDS: Self‐Rating Depression Scale
VLSQ‐8: Visual Acuity Questionnaire‐8

## 1 Introduction

Migraine is a chronic neurovascular disease characterized by episodic, moderate‐to‐severe throbbing headache accompanied by nausea, vomiting, and photophobia (Steiner et al. 2016; S. Yu et al. 2012). Migraine without aura (MwoA) accounts for approximately 75% of all migraine cases and is a significant but under‐recognized public health challenge (The Lancet Neurology 2017). It causes an enormous medical burden on society and families and affects the quality of life of patients (The Lancet Neurology 2017; Pradeep et al. 2019). Thus, understanding the pathogenesis of this disease is clinically urgent. Although neuroimaging studies have shown that migraine patients have abnormal functional connectivity in specific brain regions, such as the thalamocortex, anterior insula, amygdala, dorsal pontine, and cerebellum, as well as the anterior hypothalamus, the pathophysiological mechanism of migraine is still not fully understood.

Resting‐state functional magnetic resonance imaging (rs‐fMRI) has been widely used in neurological research, including in research on migraine (Chong et al. 2017; DaSilva et al. 2007; Demir et al. 2016). As a noninvasive neuroimaging technique, rs‐fMRI provides a valuable reference for revealing the pathophysiological changes associated with diseases (Messina et al. 2023). rs‐fMRI is of great value in exploring brain functional connectivity, network properties, and topology (Demir et al. 2016; J. Zhang et al. 2017; Gaist et al. 2018; Palm‐Meinders et al. 2017; Z. B. Yu et al. 2017). Graph theory analysis is a common method for studying topological structure changes in complex brain networks. Degree centrality (DC) is the most robust graph theory analysis method; it reflects the correlation between brain regions and the whole brain and describes the degree of nodes in the center of the brain network. An increase or decrease in DC indicates that the number of connections with a brain region increases or decreases, respectively, and that the ability to transmit information is enhanced or weakened, respectively (Y. Liu et al. 2023; Qin et al. 2023; Ruan et al. 2023). DC analysis can identify voxels with altered connections to other voxels but cannot provide detailed information about the altered connections between voxels and specific regions.

Effective connectivity (EC) can reveal causal or directional effects on different brain regions. Granger causality analysis (GCA) is an effect‐linking method for evaluating EC from time series data (Cohen Kadosh et al. 2016). GCA is used widely in depression and mental addiction diseases but is rarely used in migraine (Backman et al. 2023; Chatzigeorgiou et al. 2023; J. Chen et al. 2021). L. Zhang et al. (2022) reported reduced bilateral hypothalamic outflow to the visual cortex in migraine patients during attacks compared to migraine patients during the interictal period and in healthy controls (HCs). Zhu et al. (2021) found increased and decreased negative effects of the bilateral visual cortex and the right dorsolateral prefrontal cortex on the hippocampus, respectively. However, these GCA studies for migraine were empirically assigned seed points. However, very few studies have explored effective functional connectivity in migraine patients by combining DC with GCA.

Therefore, the difference in brain network centrality between migraine patients and controls was investigated by using the voxel‐based DC method in this study. The brain regions with different DC values between the two groups were subsequently used as seed points for GCA to evaluate the changes in functional connectivity of the whole brain in migraine patients. A deeper understanding of the pathogenesis of migraine at the level of brain function is needed.

## 2 Methods

### 2.1 Subject Selection Criteria

Two groups of subjects were recruited from Nanjing Hospital affiliated with Nanjing Medical University from May 2018 to March 2021: one group had MwoA, and the other group was HCs. The participants in both groups signed informed consent before the experiment and were informed of the examination purpose, steps, and precautions, as well as possible discomfort. Specifically, 60%(*N* = 59, MwoA = 19, HC = 40) of the participants in the current study were also included in the previous study (D. Zhang, Huang, et al. 2021).

The recruited patients were a total of 50, and all of them had episodic MwoA according to the International Classification of Headache Disorders (Third Edition, beta version; ICHD‐3 beta) and were between 18 and 50 years old. After matching for age, education background, and sex, 48 HC's subjects were finally enrolled in current study.

The exclusion criteria were as follows: (1) bad image quality; (2) MRI contraindications; (3) neuropsychological disorders; (4) a history of alcohol or drug abuse; (5) brain injuries or other neurological conditions (such as epilepsy, stroke, and physical diseases) that may affect the study results; (6) immediate family members with a headache history; and (7) use of any vasoactive medication 1 week before the scan. Meanwhile, in order to reduce the effect of hormone levels on cortical excitability, MRI scans were performed during the middle of the menstrual cycle for all female subjects except during pregnancy and lactation. The patients were headache free for at least 48 h (before and after the scan), fasted for 4 h, and were not allowed to have tobacco for 12 h or drink coffee, tea, alcohol, or cocoa before the study started. Similar to our previous study, all patients were assessed through Self‐Rating Depression Scale (SDS), Self‐Rating Anxiety Scale (SAS), Montreal Cognitive Assessment (MoCA) screening test, frequency, duration, Migraine Disability Assessment (MIDAS) questionnaire, Headache Impact Test‐6 (HIT‐6), Visual Analogue Scale (VAS), as well as Visual Acuity Questionnaire‐8 (VLSQ‐8). VLSQ‐8 contains eight questions to evaluate the presence and severity of visual photosensitivity (Y. Lin et al. 2020). All subjects did not receive preventive treatment.

### 2.2 MRI Data Acquisition

A 3.0T MRI scanner (Ingenia, Philips Medical Systems, Netherlands) equipped with an eight‐channel head coil was applied for all subjects. The subjects were supine with their head secured by foam pads and straps to reduce head movement. Ear plugs with approximately 32 dB of attenuation (Hearos Ultimate Soft Series) were used. The subjects were told to lie still and close their eyes but not to fall asleep or think about anything in particular. Transverse fMRI images were gotten by gradient echo‐planar serial scanning. The parameters were as follows: repetition time (TR) = 2000 ms, echo time (TE) = 30 ms, slices = 36, thickness = 4 mm, gap = 0 mm, field of view (FOV) = 240 mm × 240 mm, acquisition matrix = 64 × 64, and flip angle (FA) = 90°. The voxel size was 3.75 mm × 3.75 mm × 4.0 mm. The functional sequence took 8 min and 10 s. A high‐resolution T1‐weighted three‐dimensional anatomical image was obtained: TR/TE = 8.1/3.7 ms, slices = 170, thickness = 1 mm, gap = 0 mm, FA = 8°, acquisition matrix = 256 × 256, and FOV = 256 mm × 256 mm. The structural sequence lasted 5 min and 29 s. Parallel imaging was used for all scans using sensitivity coding (SENSE) technology, sense factor = 2.

### 2.3 Analysis of MRI Data

#### 2.3.1 Preprocessing

The RESTplus, a resting‐state magnetic resonance data processing tool in the MATLAB platform, was utilized to analyze the MRI data. The specific steps were as follows (H. Lin et al. 2022; Niu et al. 2022): (1) convert all the DICOM images to Neuroimaging Informatics Technology Initiative (NIFTI) format; (2) remove the first 10 time point images; (3) perform time correction through selecting the middle layer as the reference layer; (4) perform head motion correction through excluding subjects in the file‐generated standard report, with 2.5 mm or 2.5° used as the head motion threshold to delete images of subjects who did not meet the standard; (5) achieve space standardization with two‐part registration method, all images spatially corrected, and all images segmented and registered to the standard Montreal Neural Institute (MNI) template; (6) filter the signals with a filtration frequency of 0.01–0.08 Hz to exclude the interference of physiological low‐frequency signals (heartbeat or breathing) or high‐frequency random noise; and (7) remove linear drift to exclude a linear signal accumulated over time by machine heating and test fatigue and to perform regression.

#### 2.3.2 DC Analysis

According to previous methods, Pearson correlation was applied to evaluate the strength of connections between single voxels and other voxels in the template signal (Lee et al. 2019). In this study, RESTplus software was utilized to perform DC analysis on all the preprocessed functional images, and the threshold was set to 0.25 to calculate the weighted DC value. The data were normalized with the Fisher *z*‐transformation, dividing the DC values for all subjects by the whole brain average. The *z* value was spatially smoothed using isotropic full‐width and half‐height smoothing with a size of 8 mm × 8 mm × 8 mm. Smoothing can reduce the error in the brain sulcus gyrus structure after image standardization and increase the normality of the data, which is conducive to data analysis.

#### 2.3.3 GCA Analysis

Consistent with previous studies (Y. Chen et al. 2022; Huang et al. 2021), based on MNI standard spatial coordinates, the MNI peak coordinate points with significant differences in DC between the two groups were taken as seed points, and the sphere with a radius of 6 mm was adopted as the region of interest. RESTplus software was utilized to calculate the causal effects of the time series of seed points and time series of each voxel in the whole brain. The time series of the seed points is defined as time series *x*, and the time series of each voxel in the whole brain is y. Positive values of the effect connections from *x* to *y* indicate that the activity of the seed points has a causal relationship in the same direction as the activity in the whole brain, while negative values indicate the opposite direction. Each seed site was analyzed twice, with a causal effect linkage analysis from the seed site to the whole brain voxel (*x*–*y*) and a causal effect linkage analysis from the whole brain voxel to the seed site (*y*–*x*). The GCA graph is converted through Fisher *z* to obtain the *z*‐valued GCA graph.

### 2.4 Statistical Analysis

SPSS 20.0 software was utilized to perform an independent sample *t*‐test for data measurement. The results are expressed as the mean ± standard deviation, and *p* < 0.05 was considered to indicate statistical significance. The statistical analysis of DC and GCA data from the two groups was performed with RESTplus software, and the two‐sample *t*‐test was applied to analyze the data from the two groups to explore the changes in global brain functional connectivity. The Gaussian random field (GRF) multiple comparison correction method was employed to correct the results, with single voxel level *p* < 0.001 and cluster level *p* < 0.05. DC analysis of continuum size > 18 voxels and GCA analysis of continuum size > 42 voxels were performed, and the corrected *t* value was obtained. The corrected results were visualized with xjView software. Ch2 was selected as the overlay background template, and the brain regions with differences in DC and GCA between the migraine group and the HCs group were identified. To study the relationship between DC and EC and the clinical characteristics of the MwoA patients, we extracted regions with significant differences between the MwoA patients and HCs and then calculated the mean *z* values of the masks of abnormal DC regions in each subject. Spearman's correlation analysis was applied to analyze the associations between each clinical characteristic (SAS, SDS, MoCA, frequency, duration, HIT‐6, VAS, VLSQ) and the mean *z* values of aberrant DC and EC values with SPSS 19.0 (IBM Corporation, Armonk, NY, USA), and *p* < 0.05 was set as the threshold, and the values were corrected for education level, sex, and age.

## 3 RESULTS

### 3.1 Demographics and Clinical Characteristics

Table 1 shows the demographic and clinical data for all the subjects. There were no significant differences in sex distribution (*p* = 0.565), age (*p* = 0.136), and education level (*p* = 0.999) between the MwoA group and the HC group.

**TABLE 1:** Demographic characteristics and cognitive performance in MwoA patients and HCs.

Characteristic	MwoA (*n* = 50)	controls(*n* = 48)	*p* value
Age (years)	38.40 ± 9.85	41.65 ± 11.49	0.136
Education (years)	13.98 ± 2.69	13.68 ± 3.58	0.999
Sex (female/male)	32/18	28/20	0.565
MoCA score	26.12 ± 3.21	26.10 ± 2.43	0.978
SAS score	41.43 ± 10.07	NA	—
SDS score	43.30 ± 10.34	NA	—
Frequency (d/m)	4.46 ± 6.10	NA	—
Duration (hours)	15.56 ± 9.28	NA	—
HIT‐6 score	60.06 ± 7.39	NA	—
MIDAS score	16.40 ± 10.39	NA	—
Headache laterality, n (%)
Unilateral	13 (26%)	NA	—
Bilateral	21 (42%)	NA	—
Shift	16 (32%)	NA	—
VAS	5.34 ± 1.99	NA	—
Mild, *n* (%)	14	NA	—
Moderate, *n* (%)	25	NA	—
Severe, *n* (%)	11	NA	—

*Note*: Measurement data are expressed in mean and standard deviation.

Abbreviations: HC, healthy control; HIT‐6, Headache Impact Test‐6; MIDAS, Migraine Disability Assessment; MoCA, Montreal Cognitive Assessment; MwoA, migraine without aura; SAS, Self‐Rating Anxiety Scale; SDS, Self‐Rating Depression Scale; VAS, Visual Analogue Scale 0–10: mild 1–3, moderate 4–6, severe 7–10.

### 3.2 DC Analysis

**FIGURE 1:**
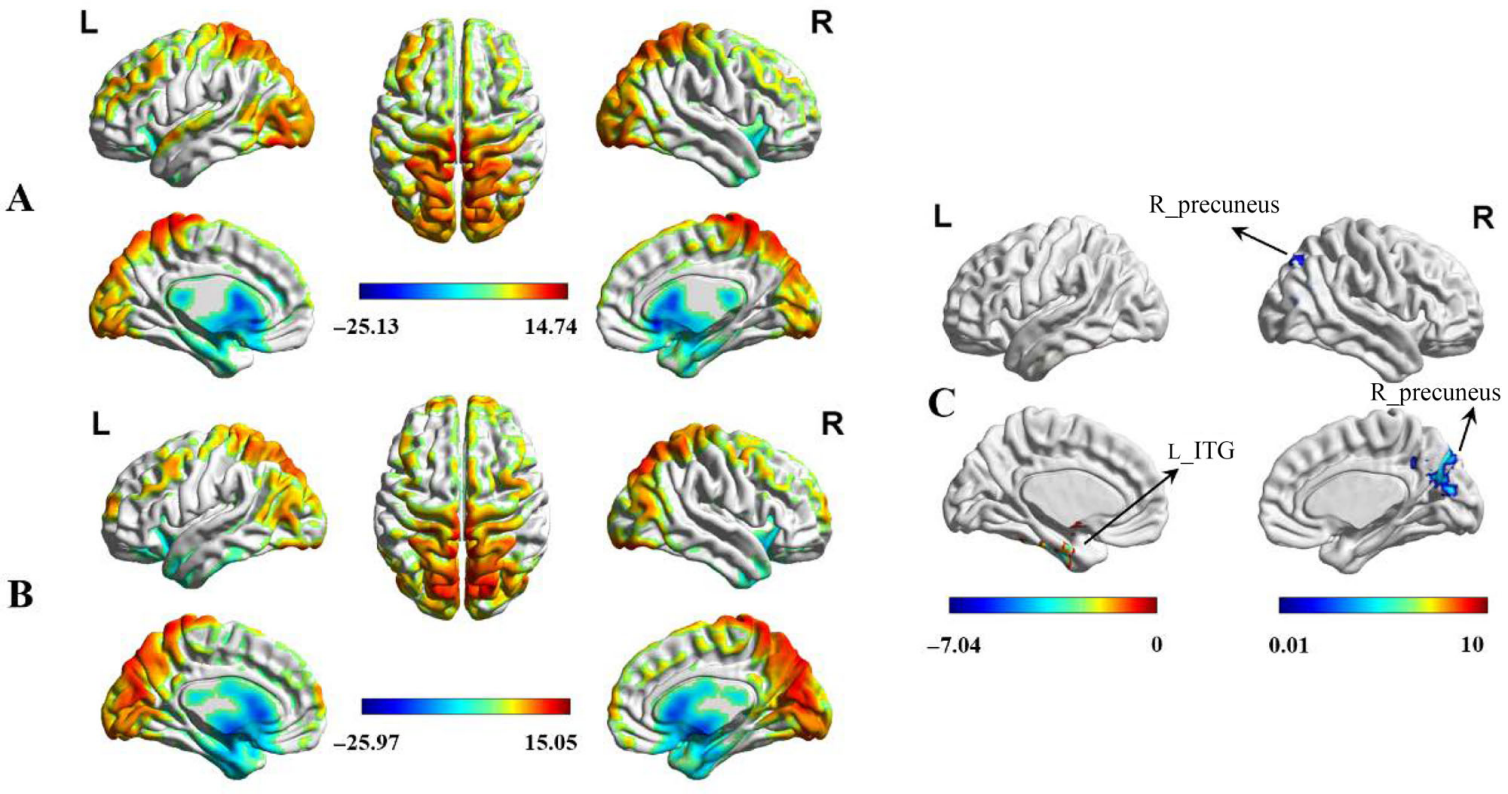
(A) Spatial distribution of the degree centrality (DC) in the healthy controls (HCs). (B) Spatial distribution of the DC in MwoA patients. (C) Significantly aberrant DC within the left inferior temporal gyrus (ITG) and right precuneus between MwoA patients and HC group. HC, healthy control; MwoA, migraine without aura.

**FIGURE 2:**
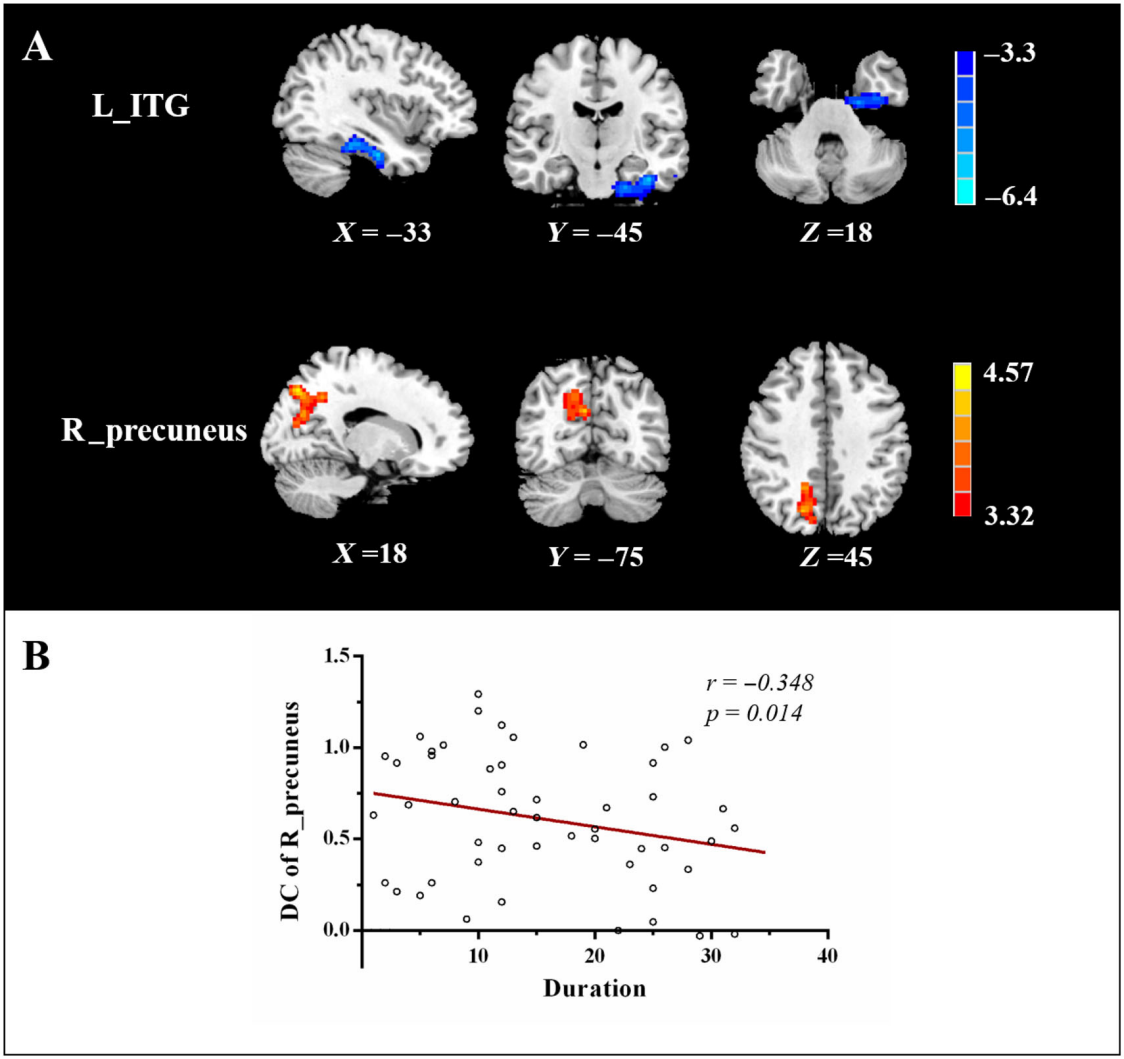
(A) Aberrant degree centrality (DC) within the left inferior temporal gyrus (ITG) and right precuneus between MwoA patients and HC group. (B) The DC values in the right precuneus negatively correlated with duration of headache in the MwoA group (*r* = −0.348, *p* = 0.014).

Figure 1A,B shows the spatial distribution of DC in the MwoA patients and HCs. The spatial distribution of the DC was highly localized in the temporal and occipital lobes, precuneus, prefrontal cortex (PFC), corpus callosum, and parahippocampal gyrus. Figures 1C and 2A show the significant group‐level differences in DC (GRF, clustering level *p <* 0.05, voxel level *p <* 0.01). Compared to the HCs, the MwoA patients showed decreased DC in the left inferior temporal gyrus (ITG) and increased DC in the right precuneus.

### 3.3 Causal Connectivity Analysis

**FIGURE 3:**
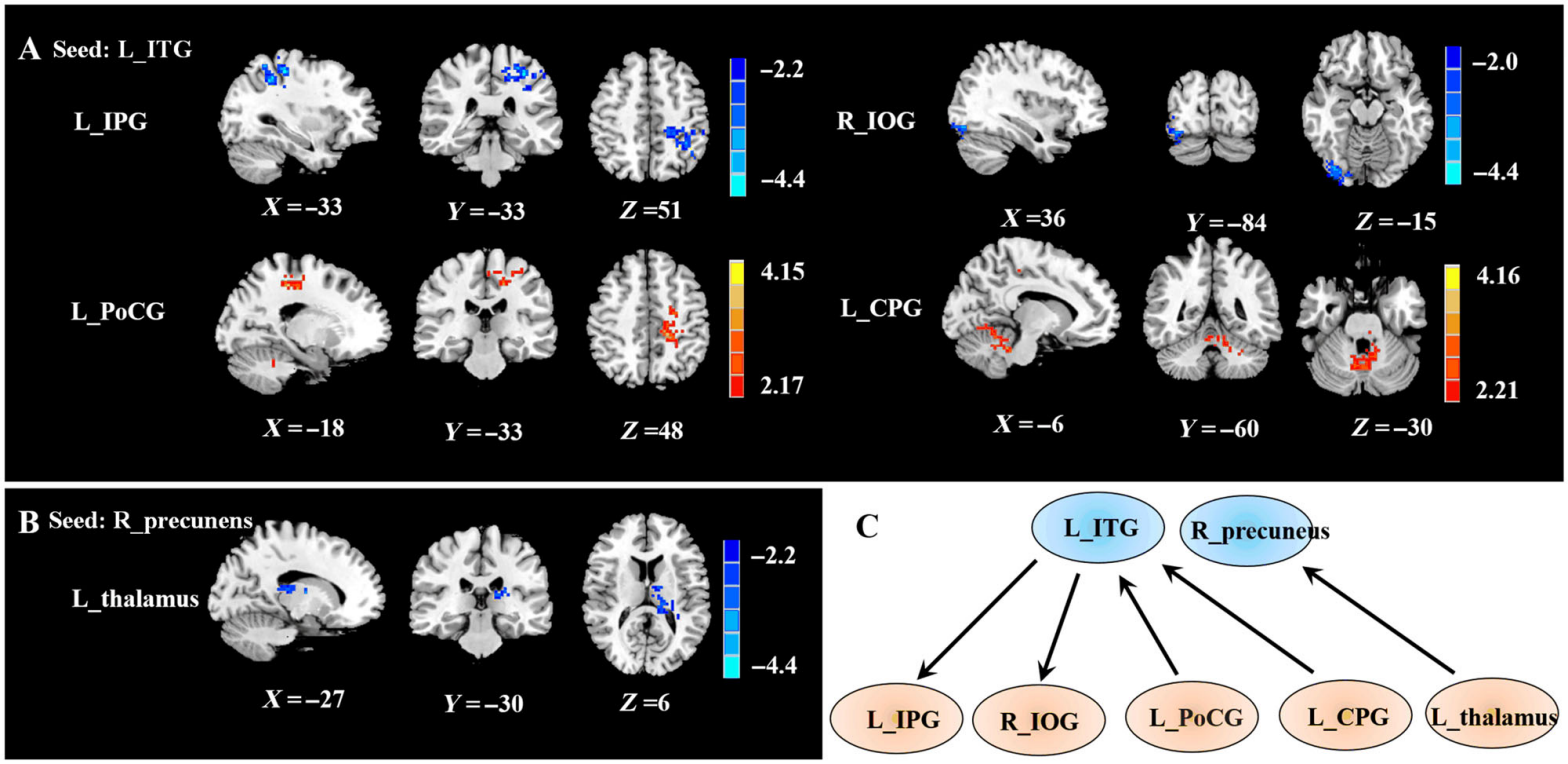
Aberrant effective connectivity in MwoA patients. (A) Decreased effective connectivity from the left ITG to the left IPG and right IOG and increased effective connectivity from the left PoCG and left CPG to the left ITG (GRF, clustering level *p <* 0.05, voxel level *p <* 0.01). (B) Decreased effective connectivity from the left thalamus to the right precuneus (GRF, clustering level *p <* 0.05, voxel level *p <* 0.01). (C) Schematic overview of changes in effective connectivity from the left ITG and right precuneus. CPG, cerebellum posterior lobe; IOG, inferior occipital gyrus; IPG, inferior parietal gyrus; MwoA, migraine without aura; PoCG, postcentral gyrus.

Compared to the HCs, the MwoA patients in the present study exhibited significantly decreased EC from the left ITG to the left inferior parietal gyrus (IPG) and right inferior occipital gyrus (IOG) as well as significantly increased EC from the left postcentral gyrus (PoCG) and left cerebellum posterior lobe (CPG) to the left ITG. Moreover, the MwoA patients exhibited decreased EC from the left thalamus to the right precuneus compared to HCs (Figure 3). However, there was no significant difference in causal inflow from the right precuneus to the rest of the brain (*p* > 0.05).

### 3.4 Correlation Results

**FIGURE 4:**
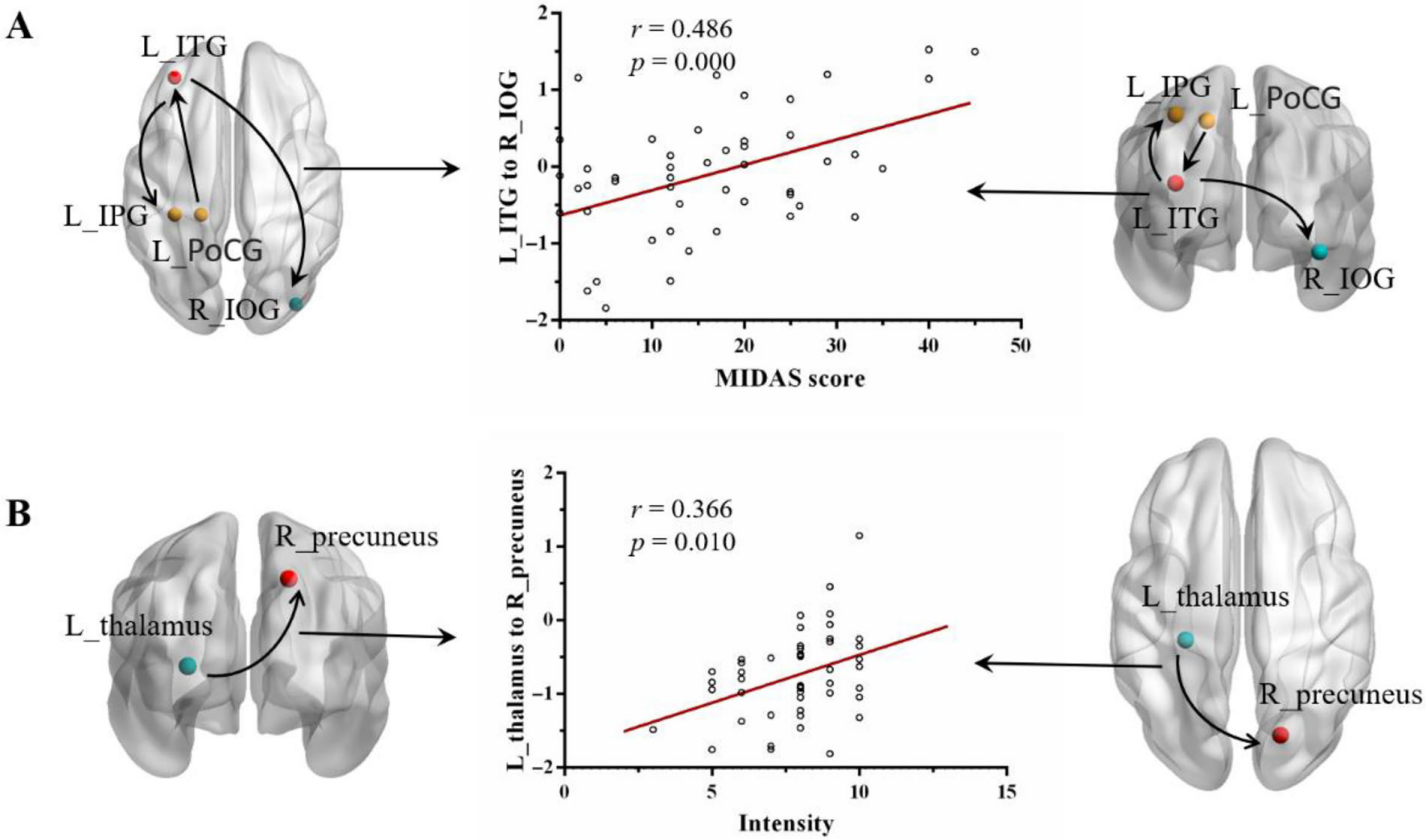
Correlations between the effective connectivity and clinical characteristic or neuropsychological performance. (A) The MIDAS scores positively correlated with the effective connectivity from the left ITG to right IOG (*r* = 0.486, *p* = 0.000). (B) The intensity of headache positively correlated with the effective connectivity from the left thalamus to right precuneus (*r* = 0.366, *p* = 0.010). IOG, inferior occipital gyrus; ITG, inferior temporal gyrus; L, left; MIDAS, Migraine Disability Assessment; R, right.

The significant correlations between DC and EC changes and clinical measures of migraine are depicted graphically in Figures 2B and 4. In the MwoA group, the DC values in the right precuneus were significantly negatively correlated with the duration of headache (*r* = −0.348, *p* = 0.014). Additionally, we found a significantly positive correlation between the MIDAS score and the EC from the left ITG to the right IOG (*r* = 0.486, *p* = 0.000), as well as between the intensity of headache and the EC from the left thalamus to the right precuneus (*r* = 0.366, *p* = 0.010).

## 4 Discussion

In this study, we explored the intrinsic functional structure of the whole brain and its relationship with clinical measures of migraine in the MwoA patients. We found that compared to the HCs, the MwoA patients exhibited (1) a decreased DC value in the left ITG and an increased DC value in the right precuneus, (2) abnormal EC between the significant cluster (left ITG and right precuneus) and several brain regions of the whole brain, and (3) changes in DC and EC were significantly associated with the relationships between the DC value in the right precuneus and duration of headache and between the EC (L_ITG to R_IOG, and L_thalamus to R_precuneus) and the clinical parameters.

ITG is associated with improved cognitive function, including deterioration of pain (Y. Lin et al. 2020). The ITG receives incoming information from the occipital lobe (Weiner and Zilles 2016; H. Zhang, Li, et al. 2021), which may be the primary region of visual processing. There is evidence that reduced cortical thickness in the ITG may lead to abnormalities in the multisensory integration of visual processing, such as in photophobia, which may cause or accompany migraine attacks (Guarnera et al. 2021). Previous studies have shown that the ITG plays a key role in interconnectivity with other multisensory cortical regions and is thought to form a multisensory integration network (Helmchen et al. 2014; Rao et al. 2023). Consistent with the findings of previous research, our study showed that migraine patients have lower ITG DC than HCs (Rao et al. 2023). Most MwoA patients exhibit photophobia and phonophobia. Since the ITG is involved mainly in visual perception, we hypothesized that it may also be associated with photophobia symptoms in MwoA patients.

The precuneus is thought to be central to a range of highly integrated tasks, such as the integration of auditory, somatosensory, and visual information, as well as the perception and transmission of pain (Cavanna and Trimble 2006; Zhe et al. 2021). One study revealed that abnormal dynamic functional network connectivity between the precuneus and the visual cortex correlated with headache frequency in migraine patients (Tu et al. 2019). In addition, a study showed that FC enhancement between the right precuneus and the left PoCG during migraine attacks was inversely correlated with headache intensity (Wei et al. 2020). In addition to functional MRI studies, EEG studies have identified abnormalities in the precuneus in migraine patients. Haehner et al. (2023) found that the precuneus was activated during the chemical sensory stimulation. The estimated current source density from the precuneus was positively correlated with the clinical score “years with migraine,” confirming the crucial role of this specific area in migraine pathophysiology. In the present study, we found that an increased DC in the precuneus was negatively correlated with headache duration. The precuneus is an important component of the DMN, and higher precuneus DC values are associated with greater ability to maintain homeostasis and, therefore, shorter headache durations.

The PoCG belongs to the primary somatosensory cortex (S1), which is involved in the perception and processing of pain signals (Rushworth et al. 2003). Functional imaging studies have shown that S1 significantly changes nerve function activity in response to exogenous pain stimuli (Bushnell et al. 1999). One study revealed higher neural activity in the bilateral PoCG in MwoA patients than in HCs (Wei et al. 2020). In addition, in rs‐fMRI studies of migraine, the FC of the PoCG has been found to change (Wei et al. 2020; Feng et al. 2022; Ke et al. 2020). Our results showed that the effective connection between the left PoCG and left ITG increased, which also explained the high sensitivity of migraine patients to sound and light.

Similar to our previous study, we found abnormal functional connectivity of the CPG (D. Zhang, Huang, et al. 2021). The CPG is abnormally activated in migraine patients compared to HCs (Zhe et al. 2021). Recently, there has been growing evidence that the cerebellum is involved not only in motor control but also in cognitive and emotional functions. The CPG mainly receives afferent information from the cerebral pontine cerebellar fasciculus and olivocerebellar fasciculus. The CPG is thought to play an inhibitory role in pain perception (Moulton et al. 2010; Stoodley and Schmahmann 2010; Timmann et al. 2010; Noseda 2022). Therefore, the increased EC from the CPG to the ITG in migraine patients may be related to the inhibition of headache perception and the maintenance of internal homeostasis.

The IPG is known to be involved in spatial discrimination and pain perception (Fassbender et al. 2004; May 2008). The IPG is involved in the pathophysiology of migraine. Many previous studies have shown abnormal changes in the structure and function of the IPG in migraine patients. Morphological studies have shown that patients with MwoA exhibit a reduction in IPG cortical thickness and gray matter volume compared to HCs (Coppola et al. 2015; Z. B. Yu et al. 2016). Graph theory analysis revealed a decrease in IPG node centrality in MwoA patients compared to HCs, indicating a decrease in information integration in this region (J. Liu et al. 2012). In addition, dysfunction of the IPG may be related to the central sensitization and hypersensitization of migraine patients to painful stimuli (Hu et al. 2019; N. Chen et al. 2015). Therefore, reduced outflow from the ITG to the left IPG may result in an inability to adequately divert attention from pain. The IOG is involved in the transmission of visual information (Wandell et al. 2005). There are direct white matter fiber bundle connections between the IOG and the ITG (Palejwala et al. 2020). Compared to those of HCs, patients with MwoA had reduced ReHo values in the IOG, which correlated with the duration of the disease (Li et al. 2020). Decreased outflow from the ITG to the IOG may lead to decreased regulatory perception of light, causing increased sensitivity to light in migraine patients. Our results showed that the MIDSA score was positively correlated with the EC from the left ITG to the right IOG, suggesting that the lower ITG to the IOG outflow has a greater impact on the lives of migraine patients.

The thalamus is an important relay station of the central nervous system that transmits various sensory signals, such as touch, pain, vision, and hearing, to the cortex and realizes the cortical–cuticular communication bridge (Dai et al. 2023). Our results showed a decrease in the EC from the thalamus to the precuneus, which was positively correlated with headache intensity. In addition, the thalamus is also an important node of the closed circuit of the trigeminal nerve‐thalamic‐cerebral cortex and mainly plays an inhibitory role in nerve activities such as pain (Ganmor et al. 2010). The EC from the thalamus to the precuneus is reduced, so this inhibitory effect is also weakened.

This study has several limitations. First, due to the small sample, the results should be considered preliminary. Second, only MwoA patients were included in this study. In the future, patients with migraine with aura will be further included in a more detailed subgroup analysis. Third, with the cross‐sectional design, it was not possible to determine whether the detected abnormalities in brain function predisposed a person to migraine or were caused by recurrent migraine attacks. In the future, we will follow up with the subjects and conduct a longitudinal study. In addition, our results cannot be generalized to patients with chronic migraine.

## 5 Conclusions

In summary, this study aimed to detect functional network connections in MwoA combining DC with GCA. The MwoA patients showed decreased DC in the left ITG and increased DC in the right precuneus. Group differences in the GCA results indicated that the ECs among the PoGG, CPG, IPG, IOG, and ITG changed. There were also abnormalities in the EC between the thalamus and the precuneus. These areas involve multisensory integration, visual transmission, perception, and processing of pain signals. Understanding the various accompanying symptoms of photophobia, photophobia, and vertigo in migraine patients can help to reveal the pathophysiological basis of these conditions. GCA results indicate that the EC among the PoGG, CPG, IPG, IOG, ITG, the thalamus, and the precuneus changed.

## Author Contributions

**Di Zhang**: formal analysis, writing–original draft. **Liyan Lu**: resources, visualization. **Xiaobin Huang**: resources. **Xiaojing Zhao**: data curation. **Yamei Zhang**: methodology. **Tong Fu**: software, visualization. **Fengfang Li**: project administration, supervision, writing–review and editing. **Xinying Wu**: methodology, project administration, supervision.

## Ethics Statement

Ethics Committee approval was obtained from the Institutional Ethics Committee of Nanjing First Hospital prior to the commencement of the study.

## Conflicts of Interest

The authors declare no conflicts of interest.

### Peer Review

The peer review history for this article is available at https://publons.com/publon/10.1002/brb3.70414

## Data Availability

The data that support the findings of this study are available from the corresponding author upon reasonable request.
